# Melanoma-intrinsic β-catenin signaling prevents T cell infiltration and anti-tumor immunity

**DOI:** 10.1186/2051-1426-2-S3-O15

**Published:** 2014-11-06

**Authors:** Stefani Spranger, Riyue Bao, Thomas Gajewski

**Affiliations:** 1 University of Chicago, Chicago, IL, USA

## 

A subset of melanoma patients has evidence for spontaneous anti-tumor immune responses and T cell infiltration into tumor sites, which has important prognostic value and is associated with clinical responses to immunotherapies. However, the molecular mechanisms explaining absence of a T cell response in the majority of patients are not defined. Analyses of human melanoma metastases by exome sequencing, gene expression profiling, and IHC have revealed that many tumors that lack a T cell signature show alterations in the Wnt/β-catenin signaling pathway. To investigate if constituently active β-catenin signaling within the tumor cells might inhibit immune responses, we utilized an inducible autochthonous mouse melanoma model driven by inducible Braf^V600E ^and PTEN-deletion, with or without inducible expression of active β-catenin. While Braf/PTEN melanomas showed presence of a modest T cell infiltrate, T cells were nearly completely eliminated in tumors expressing active β-catenin. The T cells that were present in the Braf/PTEN tumors showed an exhausted phenotype as what has been seen in transplanted tumor models, and therapeutic efficacy of combination therapy using αCTLA4 with αPD-L1 mAbs was limited to Braf/PTEN tumors. To test whether the lack of T cell infiltration was due to a defect in early T cell priming, these mice were additionally bred to inducibly express the SIY model antigen within the developing tumors. Adoptive transfer of 2C TCR Tg SIY-specific T cells revealed defective spontaneous T cell priming when tumors expressed active β-catenin. Analysis of the antigen-presenting cell compartment revealed a selective decrease in the CD103^+ ^DC subset within the tumor microenvironment, and T cell infiltration could be restored by intra-tumoral injection of FLT3-ligand-derived dendritic cells. The lack of CD103^+ ^dermal dendritic cells was associated with reduced expression of the chemokines CCL4 and CXCL1. Surprisingly we identified that tumor cells themselves as the major chemokine source in Braf/PTEN tumors, while tumors with active β-catenin signaling lacked expression of those chemokines. Therefore, our data have identified the first defined molecular pathway in tumor cells that results in defective spontaneous anti-tumor T cell responses, an observation with important implications for cancer immunotherapy.

## Consent

Written informed consent was obtained from the patient for publication of this abstract and any accompanying images. A copy of the written consent is available for review by the Editor of this journal.

